# Reward Contingencies Improve Goal-Directed Behavior by Enhancing Posterior Brain Attentional Regions and Increasing Corticostriatal Connectivity in Cocaine Addicts

**DOI:** 10.1371/journal.pone.0167400

**Published:** 2016-12-01

**Authors:** Patricia Rosell-Negre, Juan-Carlos Bustamante, Paola Fuentes-Claramonte, Víctor Costumero, Juan-José Llopis-Llacer, Alfonso Barrós-Loscertales

**Affiliations:** 1 Departamento de Psicología Básica, Clínica y Psicobiología. Universitat Jaume I, Castellón, Castelló de la Plana, Spain; 2 Departamento de Psicologia y Sociología. Universidad de Zaragoza, Zaragoza, Zaragoza, Spain; 3 FIDMAG Germanes Hospitalàries Research Foundation Barcelona, Cataluña, Spain; 4 Unidad de Conductas Adictivas, Hospital General Universitario, Consellería de Sanitat, Castellón de la Plana, Spain; Centre de neuroscience cognitive, FRANCE

## Abstract

The dopaminergic system provides the basis for the interaction between motivation and cognition. It is triggered by the possibility of obtaining rewards to initiate the neurobehavioral adaptations necessary to achieve them by directing the information from motivational circuits to cognitive and action circuits. In drug addiction, the altered dopamine (DA) modulation of the meso-cortico-limbic reward circuitry, such as the prefrontal cortex (PFC), underlies the disproportionate motivational value of drug use at the expense of other non-drug reinforcers and the user’s loss of control over his/her drug intake. We examine how the magnitude of the reward affects goal-directed processes in healthy control (HC) subjects and abstinent cocaine dependent (ACD) patients by using functional magnetic resonance imaging (fMRI) during a counting Stroop task with blocked levels of monetary incentives of different magnitudes (€0, €0.01, €0.5, €1 or €1.5). Our results showed that increasing reward magnitude enhances (1) performance facilitation in both groups; (2) left dorsolateral prefrontal cortex (DLPFC) activity in HC and left superior occipital cortex activity in ACD; and (3) left DLPFC and left putamen connectivity in ACD compared to HC. Moreover, we observed that (4) dorsal striatal and pallidum activity was associated with craving and addiction severity during the parametric increases in the monetary reward. In conclusion, the brain response to gradients in monetary value was different in HC and ACD, but both groups showed improved task performance due to the possibility of obtaining greater monetary rewards.

## Introduction

Drug addiction is characterized by an enhanced and recurring motivational value of the drug, at the expense of other non-drug reinforcers, and an impaired ability to inhibit intentional actions associated with strong desires to take the drug that result in a pathological and compulsive pattern of drug-seeking and drug-taking [1–3]. This process occupies an inordinate amount of an individual’s time and thoughts, and it persists despite adverse consequences [4–5].

During goal-directed behavior, changes in DA activity transform information about a reward into abstract cognitive decisions, which in turn are translated into specific actions taken to achieve the reward [6–11]. However, DA-mediated appetitive motivation drives behaviors that are not always under goal-directed control and can be maladaptive [6], as occurs in drug addiction [12–20]. Specifically, the improper DA modulation of the PFC via frontostriatal connections [1,3,20] implies PFC dysfunction and the user’s loss of control over the drug craving [21]. This craving is characterized by great salience and brain reactivity to cocaine-related stimuli at the expense of other appetitive stimuli [22–24], and it is considered a sign of addiction severity in drug users [22,25,26,27].

Chronic cocaine users often perform poorly on goal-directed tasks [16,27–31]. However, in spite of frontostriatal dysfunction, reward seems to behaviorally improve cognitive performance similarly in cocaine addicts and in HC [32–34] by involving the compensatory activity of other brain regions, such as the occipital, parietal or temporal lobes [16,35,36]. Unlike controls, addicts show a disruption in the ability to perceive inner motivational drives because there is no relationship between reward induced improvement and self-reported interest in the reward value [37]. Therefore, the increase in awareness of inner motivational drives and the improvement in goal-directed behavior produced by reward contingencies via brain compensation may be two of the most important points to consider in dealing with drug addiction at the psychological and neurobiological levels.

In this study, HC and ACD performed a counting Stroop task using blocked levels of monetary incentives to analyze the effects of parametric increases in monetary rewards on performance and brain activation patterns. The aims of our study include comparing HC and ACD on how the parametric increses in the monetary reward magnitude modulates: (1) the performance during goal-directed behavior (e.g., reaction time, accuracy, and response variability) and the relationship between performance and self-reported interest in the reward value; (2) the brain activation in frontal and striatal regions generally involved in Stroop performance and posterior brain regions involved in the Stroop task in ACD; (3) the connectivity between the PFC and striatal regions; and (4) the way individual differences in clinical variables such as craving or severity dependence are related to the modulation of the reward magnitude on brain function. We hypothesized that as the size of the reward contingencies increases: (1) the task performance will improve in both experimental groups, but this improvement will be related to self-reported interest in the reward value only in HC; and (2) the activity of the prefrontal regions will be deficiently modulated in ACD compared to HC, but ACD will enhance the activation of other brain regions to counteract prefrontal impairment; moreover, (3) ACD prefrontal function will be related to a different pattern of frontostriatal connectivity compared to HC; and (4) higher scores on clinical variable scales will be related to activation of the frontal and striatal regions during the reward magnitude increases.

## Materials and Methods

### Participants

Thirty-seven HC and thirty-four ACD, matched on age, sex, laterality, and educational level, participated in this study (see Table 1). On the one hand, ACD patients were recruited from the Addictive Behaviors Unit in Castellón and Sagunto (Spain) from patients who regularly visit the clinic to manage their abstinence. In addition to behavioral and/or pharmacological treatment, patients were monitored for continued abstinence with urine toxicology testing or through clinical interviews with a psychiatrist who supervised treatment. All 34 patients received the primary Axis I diagnosis of cocaine dependence according to the Diagnostic and Statistical Manual of Mental Disorders, Fourth Edition (DSM-IV). Due to the high rates of comorbidity with alcohol and other drug abuse in the patient population, patients were not excluded if they had abused other drugs or alcohol prior to the onset of their cocaine abstinence. On the other hand, the HC group was recruited by placing posters in public places such as town halls and universities, and through word of mouth. A previous interview collected past and current drug use data and ensured that HC had no history of psychoactive substance dependence or abuse. Exclusion criteria for HC and ACD were as follows: 1) Any major psychiatric illness; 2) Head trauma resulting in loss of consciousness for longer than 30 min; 3) Presence of any past or current brain pathology; 4) The presence of any contraindications to an MRI environment. Before the scanning session, the ACD filled in two addiction severity-related scales: the Cocaine Selective Severity Assessment (CSSA, [38]) and the Spanish version of the Severity Dependence Scale (SDS, [39]). Furthermore, patients were screened on their craving-related score, based on the 12-item Spanish version of the Cocaine Craving Questionnaire-General (SCCQ-G-12, [40]) (see Table 1). All the participants received information about the nature of the research, provided written informed consent prior to participating in the study, and received a monetary award for their participation depending on their performance on the task. The institutional Review Board of the Universitat Jaume I (Castellón, east Spain) approved this study.

**Table 1:** Demographic variables for all study participants and clinical variables for ACD.

	HC (N = 37)	ACD (N = 34)
**Age (years)**	36.89 (8.937)	36.65 (7.118)
**Sex: men/women**	31/6	30/4
**Laterality: right/left-handed/ambidextrous**	37/0	31/1/2
**Education (years)**	11.95 (2.808)	10.94 (3.829)
**Duration of current abstinence (months)**		10.16 (14.639); range (0–50)
**Duration of cocaine use (years)**		15.147 (6.387); range(3–30)
**Age of onset of cocaine use**		19.853 (4.781); range (14–34)
**CSSA**		44 (20.999); range (0–81)
**SDS**		9.765 (2.511); range (5–15)
**SCCQ-G-12**		2.628 (1.562); range (0.33–5.92)
**Psychotherapy: yes/no**		19/15

HC: Healthy Controls; ACD: Abstinent Cocaine Dependents; WAIS-III: Matrix Reasoning test; CSSA: Cocaine Selective Severity Assessment; SDS: Severity of Dependence Scale; SCCQ-N-10: CCQ-N-10 in its Spanish adaptation.

### Task design

**Fig 1:**
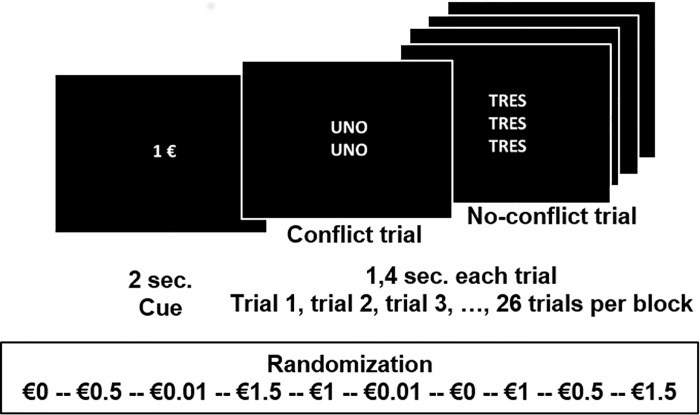
Schematic representation of the counting Stroop task in an fMRI block-design. “sec.”, seconds; €, euros. The figure represents two examples of trials (a no-conflict trial and a conflict trial) included in a €1 block, which is one of the ten randomized blocks.

We scanned all the participants while they performed a counting Stroop task with reward contingencies (see Fig 1). The task was an adaptation from an earlier study [41]. Participants viewed sets of one to four identical number words that appeared on the screen in each trial during the entire paradigm: “one”, “two”, “three” and “four” (“uno”, “dos”, “tres” “cuatro” in Spanish). We instructed them to respond as quickly as possible by pressing a button on a keypad containing two buttons for each hand (Response Grips, NordicNeuroLab, Norway), coinciding with the number of words in each set. Inside the scanner, subjects performed 10 functional blocks consisting of 26 trials each, yielding a total of 260 trials. Each block included 5 congruent trials (15%, n = 40), where the number of words agreed with the number word, (e.g., "two" "two", response: two). On the incongruent trials (85%, n = 220, 21 in each block), the number of words was discordant with the number word, (e.g., "one" "one", response: two). Congruent trials were intermixed among the incongruent trials to minimize strategy effects and increase interference [42]. In addition, we applied a linear parametric approach with five “reward” conditions corresponding to the possibility of obtaining different monetary incomes (€0, €0.01, €0.5, 1€, €1.5) for correct task performance. Participants repeated each condition in two random blocks within a single run. Our goal was to analyze the effects of reward on goal-directed behavior and, to this end, we examined the effects of reward increases during the performance of a blocked interference condition rather than during a passive conditioning task. We applied a parametric design to avoid the shortcomings of using a neutral or congruent condition for cognitive subtraction. Instead, we included a fixation point shown for 7200 milliseconds (msec.) between each task block as the baseline, which also precluded carryover effects.

The fMRI paradigm started with a baseline block. After this first baseline block and in the following ones, task blocks started with a cue (C) that appeared and lasted 2 seconds (sec.) The cue informed the participants about the amount of money (€0, €0.01, €0.5, €1 or €1.5) they could win for each correct response within each corresponding Stroop block. After each cue, we displayed a fixation point for 500 msec. just to maintain participants’ attention. Afterward, we presented 26 trials, each lasting 1000 msec. with an inter-trial interval of 400 msec. The duration of each Stroop block was 46.7 sec., whereas total task duration was 7 minutes and 41 sec. Participants did not receive feedback on their performance at any time during the task, only at the end. The stimuli presented throughout all the trials were white on a black background (resolution of 800x600 pixels). We controlled the stimulus presentation with the Presentation software (http://www.neurobs.com).

Before they entered the scanner, we instructed all the participants about the task by reading identical instructions. The instructions explained that the participants had to respond to the number of words that appeared on the screen, not to the number word. We also told them that before each set of trials, they would see an informative cue that determined the amount of money they would receive for each correct response obtained in each set of trials (e.g., €0.50), and they would receive a monetary reward when participation ended, based on their task performance. Thus, their main goal was to win as much money as possible. After receiving the instructions, the participants completed a practice version with 90 trials to minimize practice effects and get used to matching responses to the button to press. Upon task completion, we asked 67 subjects (34 HCs and 33 ACDs) to rate their self-reported interest in the reward value for the five monetary amounts on a visual analogue scale (range: 0 to 7, boring to interesting, respectively).

### fMRI Acquisition

We acquired blood oxygenation level-dependent (BOLD) fMRI data in a 1.5-Teslas Siemens Avanto (Erlangen, Germany). We helped subjects to enter the MRI scanner and lie in a supine position. We immobilized their heads with cushions to reduce motion artifacts. We presented the stimuli via MRI-compatible goggles, and we used a response system to control performance during the scanning session (Response grips, NordicNeuroLab). We obtained functional scans using a gradient-echo T2*-weighted echo-planar MR sequence (TR = 2000ms; TE = 48 ms; matrix = 64 x 64, voxel size = 3.5 x 3.5 x 4 mm, flip angle = 90°, 4.5-mm thickness, slice gap of 0.5 mm). We acquired 24 interleaved axial slices parallel to the hippocampi and covering the entire brain. Prior to the functional MRI sequences, we acquired structural images using a high-resolution T1-weighted sequence with TR / TE = 2200 / 3.849 ms, FOV = 224 mm, matrix = 256 x 256 x 160, voxel size = 1 x 1 x 1 mm, which facilitated the localization and co-registration of the functional data.

### fMRI preprocessing

We preprocessed and analyzed the data with the SPM8 software package (Statistical Parametric Mapping 8; Wellcome Department of Imaging Neuroscience; http://www.fil.ion.ucl.ac.uk/spm), as implemented in MATLAB R2007a (Mathworks, Inc., Natick, MA, USA). Preprocessing first included the realignment of each scan per individual to the first scan to correct motion-related artefacts (movement parameters never exceeded 2 mm of translation or 2 degrees of rotation in any direction for any participant). Second, the normalization to a standard EPI template was carried out in accordance with the Montreal Neurological Institute (MNI) template by applying an affine transformation followed by nonlinear deformation, and using the basic functions defined in the SPM program. We applied the computed transformation parameters to all the functional images by interpolating them to a final voxel size of 3 x 3 x 3 mm. Finally, we spatially smoothed the images with an 8 x 8 x 8 mm (Full Width at Half Maximum; FWHM) Gaussian kernel.

### Statistical analyses

#### Behavioral analysis

Three variables related to task performance were analyzed: mean reaction time (RT) on correct responses, error rate (%), and RT variability. We measured RT variability as within-subject RT standard deviation (SDrt), as in previous studies [43,44]. We did not consider the analysis of the congruency effect because the small number of these trials and their inclusion in blocks with a higher proportion of incongruent trials may lead to unreliable estimations of the congruency effect [45–47]. Therefore, we focused on the incongruent trials. In order to compare HC and ACD on the effect of parametric increases in the monetary reward magnitude on RT, error rate and SDrt, we conducted three 5x2 mixed-design ANOVAs, including the within-subject factor Reward (€0, €0.01, €0.5, €1 or €1.5) and the between-subject factor Group (HC, ACD). Moreover, we analyzed the relationship between self-reported interest in the reward value and the performance variables, for HC and ACD separately, by correlating the self-reported interest in the reward value for each reward magnitude with the respective individual RT, error rate, and SDrt (e.g., self-reported interest in reward value for €1 correlated with RT for the €1 condition). We also tested for between-group differences in these correlation coefficients by means of Fisher’s r-to-z transformation, applying the Bonferroni correction for multiple comparisons. Based on this method, we divided the a priori selected threshold of p < .05 by the number of within variables group comparisons (k = 5), which stabilized statistical levels as significant if less than .01. We carried out these analyses using SPSS v.20 (Statistical Package for Social Sciences; SPSS Inc., Chicago, IL, USA).

#### FMRI data analysis

We performed statistical analyses following the General Lineal Model (GLM) [48]. In a block design analysis, we modeled each participant’s preprocessed time series under different conditions, using a boxcar function convolved with the hemodynamic response function. The model included seven regressors: five regressors that modeled the Stroop blocks for each monetary condition separately (€0, €0.01, €0.5, €1 or €1.5), one regressor that modeled the baseline condition (“*” for 7200 msec.), and another regressor that modeled all the informative cues. We also removed intrinsic autocorrelations by using a high-pass filter with a cut-off frequency of 128 Hz, which eliminated low-frequency components. Finally, we included the motion parameters for each subject’s realignment correction in the model as “nuisance” variables.

We generated statistical contrast images to obtain neural regions sensitive to reward gradients while performing the Stroop task, by subtracting the €0 condition from the rest of the reward magnitudes (R1 = “€0.01 *vs*. 0€”; R2 = “€0.5 *vs*. €0”; R3 = “€1 *vs*. €0”; R4 = “€1.5 *vs*. €0”). For a second-level analysis (random effects), we calculated a 4x2 mixed-design ANOVA, including the within-subject factor Reward (R1, R2, R3, R4) and the between-subject factor Group (HC, ACD) to compare HC and ACD on how parametric increases in the size of the monetary reward modulate brain activity, using the parameter estimates for the four conditions. For this parametric analysis of reward magnitudes, we fitted a parametric contrast from R1 to R4 [–2–1 1 2] as a linear signal increase from R1 to R4. Then, in order to evaluate the brain areas that showed a higher parametric modulation in HC than in ACD, we fitted the contrast [–2–1 1 2 2 1–1–2], taking into account that the first four levels pertain to the HC group, and the latter four to the ACD group. By contrast, to test the brain areas where ACD showed a higher parametric modulation than HC, we fitted the contrast [2 1–1–2–2–1 1 2].

#### Psychophysiological interactions

The PPI analysis determines the regions whose time series of activation exhibit significant covariance with the seed [49]. PPI is a context-dependent connectivity measure used to explain the regional activity of other brain regions in terms of the interaction between responses in a seed region and a cognitive process [50]. We carried out a PPI analysis using the generalized PPI toolbox (gPPI; http://www.nitrc.org/projects/gppi;[51]). For each participant and task condition (psychological variables) in the gPPI, we calculated an interaction term using the de-convolved activity of the seed region and the separate regressors that represented the task conditions. Then we re-convolved the calculated interaction terms with the hemodynamic response function [52] and included them in a GLM model, along with the time course of the seed region, the regressors modeling the task conditions, and the motion parameters. In the present study, we planned to perform a gPPI for each brain area in the PFC that showed significant differences between groups in parametric modulation as a function of the reward magnitude (see the Results section). After generating the model, we performed contrast images using the beta-weights for the interaction terms. Specifically, we generated statistical contrast images by subtracting the €0 condition from the rest of the reward magnitudes (once again, R1, R2, R3, R4) to test which regions changed their task-related connectivity with the seed as a function of the monetary reward. We performed a 4x2 mixed-design ANOVA, including the within-subject factor Reward (R1, R2, R3, R4) and the between-subject factor Group (HC, ACD) in order to compare HC and ACD on the way parametric increases in monetary reward magnitude modulate the connectivity between the prefrontal and striatal regions, and we fitted the same contrast as in the fMRI analysis to test between-group differences.

#### Regression analysis

Regression analyses were utilized to investigate the relationship between individual scores on the CSSA, SDS and SCCQ-N-10 questionnaires and brain activation during reward magnitude increases.

#### Region-of-interest analysis

We focused our analyses on regions of interest (ROIs) for two reasons: to analyze the brain regions previously associated with interference task performance in cocaine addiction, and to maximize statistical power. Following Moeller et al., [53], we defined the bilateral dorsolateral prefrontal cortex (DLPFC) (x, y, z MNI coordinates x = 44, y = 22, z = 11/-44, 21, 11) and bilateral anterior cingulate cortex (6, 23, 39/-6, 23, 38), as in the Leung et al. [54] study, which used the color-word task, the most widely employed Stroop task in studies evaluating the effects of cocaine addiction [19,53,55–60]. On the other hand, the only previous study that evaluates interference resolution during a counting Stroop task [41] found differences between HC and ACD in the right inferior frontal gyrus (45, 17,-8), right inferior parietal gyrus (53, -39, 38), and right superior temporal gyrus (53, -41, 2); thus, we also defined these areas as ROIs. We also defined posterior brain regions such as the left superior occipital cortex (-42, -88, 30), left inferior parietal cortex (-28, -38, 54), and bilateral fusiform gyrus (46, -58, -30/-24, -2, -44), based on previous task interference related effects [61–63]. We drew the ROI masks according to the Automatic Atlas Labeling from the WFU-PickAtlas [64], using a 10-mm-radius sphere centered at the peak voxel of each cluster. We also defined the bilateral caudate, putamen, and pallidum as ROIs, selected from pre-established regions of the Pickatlas toolbox, due to their involvement in the reward dopaminergic system [9,10,65,66], cognitive control and action [11,67–70], and cocaine addiction [1,32,71–73] in association with prefrontal areas [32].

All previous analyses, including parametric effects, psychophysiological interactions, and regression analyses, were based on previously defined ROIs, in which significant effects were corrected for multiple comparisons by applying a threshold at a voxel-wise corrected level (FWE at p < .05) for each ROI independently. Furthermore, we ran post-hoc analyses to examine what drove the parametric effects in all previous analyses (e.g., functional parametric effects, psychophysiological interactions, and regression analyses), obtaining a more thorough picture of what was driving the effects. However, these analyses should not be considered for statistical inference as they were biased estimates of the effect because the ROIs were non-independent but based on the parametric contrast [74,75]. In order to perform these ROI analyses, we extracted the signal from within the ROIs. Thus, we extracted the eigenvariate values from the ROIs, as implemented in SPM8. It should be noted that eigenvariate values are not the same as mean values. For the sake of reducing response heterogeneity within the cluster, eigenvariate values provide an eigenvector that downweights atypical voxels within the voxels that pass the original threshold, flexibly combining functional (e.g., statistical contrast) and anatomical (e.g., ROI coordinates) constraints [76].

## Results

### Behavioral results

**Fig 2:**
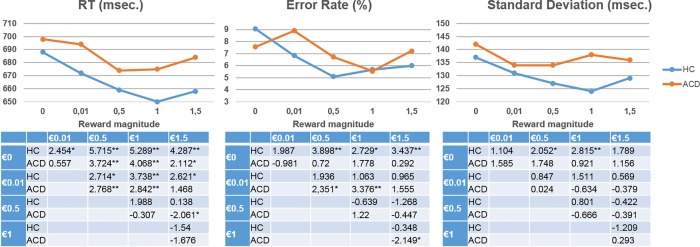
Graphical representation of performance on the different behavioral variables; and below, pairwise comparisons between reward magnitudes within each group. The results in the tables under the scatterplots show the pairwise comparison t values (* = p < .05 and ** = p < .01). msec., milliseconds; SDrt, reaction time Standard Deviation; RT, reaction time; HC, Healthy Control; ACD, Abstinent Cocaine Dependent; €, euro.

The first goal of the current study was to test whether the magnitude of the reward contingencies increased task performance similarly in HC and ACD. Table 2 and Fig 2 summarize the behavioral results. The ANOVAs showed a main effect of reward on RT (F(4,66) = 14.991; p < .001) and error rate (F(4,66) = 4.977; p = .001) and a trend toward significance for SDrt (F(4,66) = 2.213; p = .077), with no group effect or interaction (p>0.1). In spite of the lack of behavioral differences between the groups, Table 2 and Fig 2 revealed that the error rate did not behave exactly the same way for HC and ACD. In order to explore these results, we performed a post-hoc analysis by making pairwise comparisons between groups for the monetary differentials (e.g., €0 vs €0.01). The results showed significant differences between groups for the monetary differentials €0 vs €0.01 (t(69) = -2.038; p = .045) and €0 vs €0.05 (t(69) = -2.036; p = .046).

**Table 2:** Incongruent trial means (standard deviations) of behavioral variables of interest for each reward magnitude in each experimental group.

	Experimental group	€0	€0.01	€0.5	€1	€1.5
**RT(msec.)**	HC	688	672	659	650	658
		(88)	(85)	(84)	(79)	(78)
	ACD	698	694	674	675	684
		(65)	(67)	(61)	(59)	(66)
**Error rate (%)**	HC	9.073	6.821	5.084	5.663	5.985
		(6.915)	(7.321)	(5.388)	(6.258)	(6.138)
	ACD	7.563	8.894	6.723	5.532	7.213
		(6.873)	(6.349)	(5.757)	(4.007)	(5.059)
**SDrt (msec.)**	HC	137	131	127	124	129
		(26)	(26)	(28)	(24)	(26)
	ACD	142	134	134	138	136
		(26)	(27)	(27)	(25)*	(27)
**Self-reported interest in reward value**	HC	.618	1.177	3.147	4.088	4.559
		(1.577)	(1.977)	(2.35)	(2.778)	(2.894)
	ACD	1.333	1.788	3.273	3.546	3.818
		(2.483)	(2.522)	(2.415)	(2.526)	(2.675)

*p<0.05 for significant differences between groups

RT, Reaction Time; msec., milliseconds, SDrt, reaction time Standard Deviation; €, euro; HC, Healthy Control; ACD, Abstinent Cocaine Dependent.

**Fig 3:**
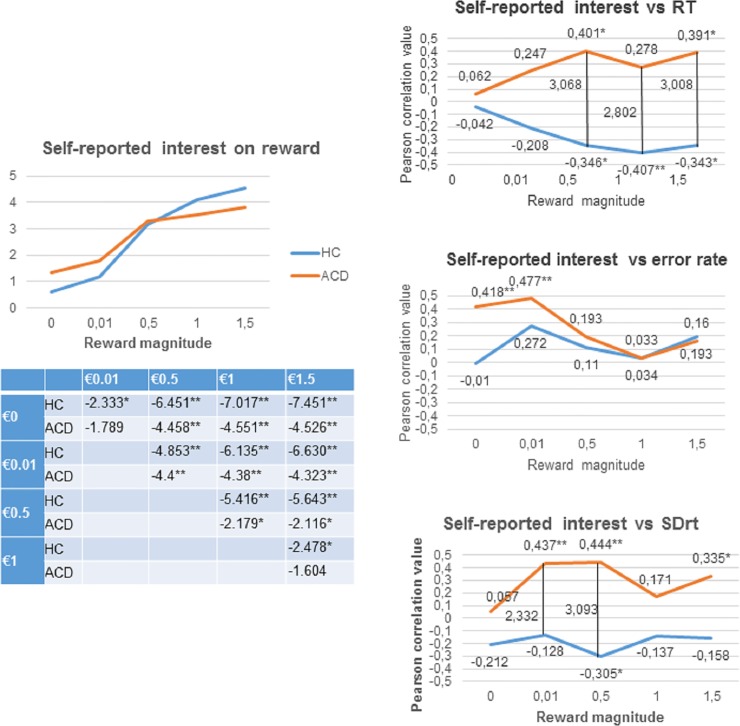
Graphical representation of associations between self-reported interest in reward value and performance, and between-group differences in these associations. The results in the left-hand table under the scatterplot shows the pairwise comparison t values (* = p < .05 and ** = p < .01) between conditions within groups. The results in the graphics on the right represent the Pearson correlation values between self-reported interest in the reward value and each behavioral variable of interest for each reward magnitude. The “*” represents significant (p < .05) and “**” represents significant (p < .01) in correlation coefficients. The numbers next to the vertical lines indicate the statistically significant between-group z-score after Bonferroni correction. RT, reaction time; SDrt, reaction time Standard Deviation; HC, Healthy Control; ACD, Abstinent Cocaine Dependent; €, euro.

The first goal of our study also included assessing whether performance improvement and self-reported interest in the reward value were related in HC but not in ACD. Self-reported interest in the reward value (34 HCs and 33 ACDs) was similar for both groups (we observed a main effect of reward condition (F(4,62) = 17.259; p < .001), without group effects (F(4,62) = 2.349; p>.05, or interactions (p>0.1)). As Fig 3 shows, RT and SDrt associations with self-reported interest were negative for HC, but positive for ACD. In order to explore whether this difference was statistically significant, we performed post-hoc between-group comparisons in correlation coefficients using Fisher´s Z transformation [77], applying the Bonferroni correction for multiple comparisons. Fig 3 shows that the correlation coefficients between groups were different (p < .05) for all the reward conditions, except €0.01, related to RT, and the €0.01 and €0.5 reward conditions, related to SDrt (see Supplementary Material S1 Fig for raw RT and SDrt data).

### Functional results

**Fig 4:**
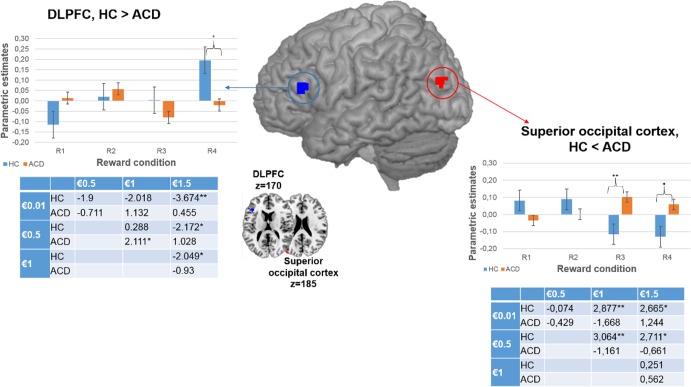
Brain regions showing parametric modulation by reward magnitude in HC>ACD and HC<ACD. The color bar represents the parameter estimates of the parametric contrast (R1<R2<R3<R4) = -2–1 1 2 for each reward magnitude. Error bars show within-subject standard error. The results in the tables show the pairwise comparison t values (* = p < .05 and ** = p < .01). DLPFC, dorsolateral prefrontal cortex; HC, Healthy Control; ACD, Abstinent Cocaine Dependent; €, euro. R1 = “€0.01 vs. €0”; R2 = “€0.5 vs. €0”; R3 = “€1 vs. €0”; R4 = “€1.5 vs. €0”

The second goal of the current study was to test whether the magnitude of the reward contingency differently modulates the activity of prefrontal regions in ACD compared to HC, although ACD show a compensatory brain activation in other brain regions to counteract prefrontal impairment. As Table 3 shows, we observed that HC showed greater activation than ACD in the left DLPFC, whereas ACD showed greater activation than HC in the left superior occipital cortex when we analyzed the parametric reward effect (R1<R2<R3<R4) (FWE at p<0.05). The rest of the ROIs did not show any significant differences between the HC and ACD groups. For illustration purposes, we plotted the parametric effect (R1<R2<R3<R4) by extracting the eigenvalues for each separate monetary condition (R1, R2, R3, R4) and for each group, from the suprathreshold voxels in the DLPFC and superior occipital cortex (see Fig 4). The beta-weights were analyzed using the SPSS software package, v.20 by performing the following analyses for each region: (1) a 4x2 mixed-design ANOVA including the within-subject factor Reward (R1, R2, R3, R4) for each group; (2) within groups pairwise comparisons between reward conditions; and (3) pairwise comparisons between groups for each reward condition (e.g., €0.5 for HC vs €0.5 for ACD). It should be pointed out that these post-hoc analyses were run to examine what drove the parametric effects, obtaining a more thorough picture of what is driving the effect. However, they should not be considered for statistical inference, as they are biased estimates of the effects because the ROIs were non-independent but based on the parametric contrast [74,75]. For the DLPFC, we observed that while HC showed a parametric effect of reward (F(3,34) = 5.058; p = 0.005), ACD did not show this effect (F(3,31) = 1.504; p = 0.233). For the superior occipital cortex, we observed again that HC showed a parametric effect of reward (F(3,34) = 6.048; p = 0.002), but ACD did not show this effect (F(3,31) = 1.003; p = 0.404). Within groups pairwise comparisons between reward conditions are depicted in Fig 4.

**Table 3:** Functional results (p = .05 family-wise error (FWE) cluster corrected).

		*Brain region*	*MNI coordinates*			*Volume (mm*^*3*^*)*	*Z score*
*Parametric effect; HC>ACD*		Left DLPFC	-42	26	16	567	3.47
*Parametric effect; ACD>HC*		Left superior occipital cortex	-39	-85	22	270	3.43
*Left DLPFC connectivity during parametric modulation; ACD>HC*		Left putamen	-27	-4	-8	243	4.09
*Regression*	*Negative correlation of SCQ-G score with parametric effect*	Left caudate	-9	2	19	351	3.75
	*Positive correlation of CSSA score with parametric effect*	Right pallidum	15	-1	-5	243	3.27

DLPFC, dorsolateral prefrontal cortex; MNI, Montreal Neurologic Institute

#### Psychophysiological results

**Fig 5:**
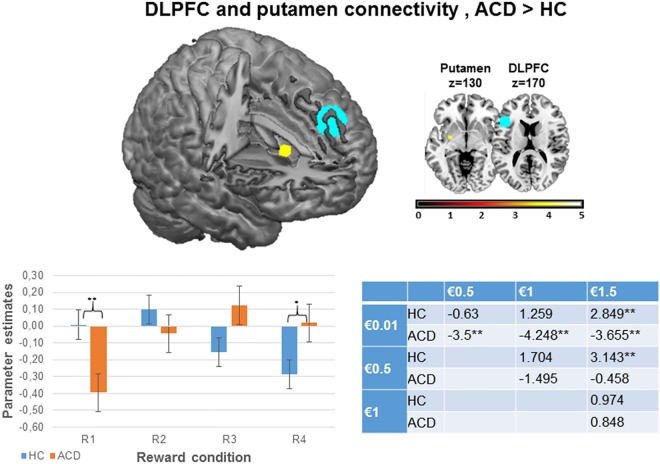
Brain regions showing changes in connectivity during parametric modulation by reward magnitude (R1<R2<R3<R4) in ACD>HC; and parameter estimates for each magnitude. DLPFC pale blue color inside the brain represents the seed region used for PPI analysis, whereas the putamen yellow color represents the color scale identified in the bar plot representing the z-values. In the graphical representation below the images, the color bar represents the parameter estimates of the parametric contrast (R1<R2<R3<R4) = -2–1 1 2 for each reward magnitude. Error bars show within-subject standard error. The results inside the table show the pairwise comparisons t values (* = p < .05 and ** = p < .01). DLPFC, dorsolateral prefrontal cortex; HC, Healthy Control; ACD, Abstinent Cocaine Dependent; €, euro. R1 = “€0.01 vs. €0”; R2 = “€0.5 vs. €0”; R3 = “€1 vs. €0”; R4 = “€1.5 vs. €0”

The third goal of the current study was to test whether ACD prefrontal impairment was related to an altered frontostriatal connectivity pattern, as previous studies have suggested [32,33]. We computed a psychophysiological analysis using the left DLPFC as a seed region because it was the only area that showed between-group differences in sensitivity to reward gradients in the PFC. Then, we ran a gPPI analysis using the same ROI that we used for the previous functional analyses in this area. As Table 3 shows, we observed greater connectivity between the left DLPFC and the left putamen in ACD than in HC when we analyzed the parametric effect (R1<R2<R3<R4) (FWE at p<0.05). For illustration purposes, we plotted the parametric effect (R1<R2<R3<R4) by extracting the eigenvalues for each separate monetary condition (R1, R2, R3, R4) and for each group, from the suprathreshold voxels in the putamen (see Fig 5). The beta-weights were analyzed using the SPSS software package, v.20, by performing the following analyses: (1) a 4x2 mixed-design ANOVA including the within-subject factor Reward (R1, R2, R3, R4) for each group; (2) within-group pairwise comparisons between reward conditions; and (3) pairwise comparisons between experimental groups for each reward condition. Again, these post-hoc analyses were run to examine what drove the parametric effects, obtaining a more thorough picture of what is driving the effect, but they should not be considered for statistical inference. We observed that both HC and ACD showed a parametric effect of reward on left DLPFC and left putamen connectivity (F(3,34) = 5.092; p = .005 for HC; and F(3,31) = 7.716; p = .001 for ACD); within-group pairwise comparisons between reward conditions are displayed in Fig 5. Finally, we performed a post-hoc gPPI analysis of the superior occipital and the putamen, in order to test the specificity of the frontostriatal connectivity, which was not significant (p>0.1).

#### Regression results

**Fig 6:**
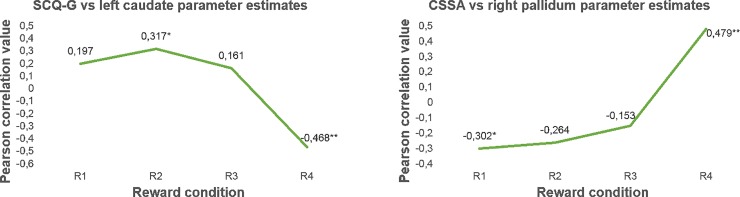
Graphical representation of the relation between CSSA (left) and SCCQ-G-12 (right) and striatal and pallidum activity for each separate reward condition. The results in the graphics represent the Pearson correlation values (* = p < .05 and ** = p < .01)

Our last goal was to test whether higher scores on clinical variable scales were related to activation of frontal and striatal parametric modulation of reward magnitude. We obtained a negative relationship between left caudate activity and SCCQ-G-12 scores (FWE at p<0.05; r(34) = -0.639, p< .001). The correlation between CSSA scores and the right pallidum was positive (FWE at p<0.05; r(34) = 0.529, p = .001). For illustration purposes, we plotted the parametric effect (R1<R2<R3<R4) by extracting the eigenvalues for each separate monetary condition (R1, R2, R3, R4), from the suprathreshold voxels in the caudate and pallidum (see Fig 6). Again, these post-hoc analyses were run to examine what drove the parametric effects, obtaining a more thorough picture of what is driving the effect, but they should not be considered for statistical inference.

## Discussion

In our study, we examined whether increasing the monetary reward magnitude modulated the goal-directed behavior and neural activation in HC and ACD during a counting Stroop condition. At the behavioral level, our study showed that the monetary reward reduces the RT and error rates similarly in both groups. In the absence of between group differences, we observed that self-reported interest in the reward value was related to RT and SDrt in both groups, but these correlations were negative for HC and positive for ACD. At the brain level, increasing the reward magnitude increased left DLPFC activation in HC compared to ACD, whereas it increased left superior occipital cortex activation in ACD compared to HC. In addition, increasing the reward magnitude strengthened the connectivity between the left DLPFC and the left putamen more in ACD than in HC. Finally, high individual craving scores were negatively associated with the left caudate, and dependence severity scores were positively related to the right pallidum during parametric reward increases. Therefore, the present study shows differences between HC and ACD in the regional brain activation and connectivity involving the frontostriatal circuitry and other areas, as a function of increasing the monetary reward magnitudes and clinical variables, with no behavioral differences between the groups’ performance, but within-group enhanced behavioral performance under reward conditions.

### Impact of reward on performance and its relation with self-reported interest on reward value

The behavioral results confirm the enhanced task performance in both groups under monetary reward contingencies in the absence of significant differences between HC and ACD in performance improvement; that is, both groups decreased their RTs and error rate as the monetary reward increased. Reward-associated enhanced performance in our study agrees with previous reports that evaluated the effects of a monetary reward in cocaine addicts and healthy controls during cognitive control tasks such as drug Stroop [32], spatial learning [33], and decision making [34]. Therefore, although drug addiction is characterized by an enhanced motivational value of the drug at the expense of other reinforcers and impaired cognitive control [1], our results and previous ones suggest that cocaine addicts’ goal-directed behavior benefits from an immediate monetary reward contingency. Regarding the relationship between self-reported interest and behavioral improvement, similarly to Goldstein et al. [37], we did not observe a group effect or an interaction effect on self-reported interest. However, we obtained between-group differences when comparing the correlation coefficients, with a trend toward showing the opposite pattern of correlations for HC and ACD between self-reported interest and RT and SDrt. These results might be interpreted as additional evidence for reward threshold elevations or reward sensitivity decreases in cocaine addicts [78,79]. Otherwise, these results can be explained in the context of secondary reinforcing effects of money in rewarding drug procurement, associated with money availability [80–82]. We speculate about whether the fact that increases in the monetary reward magnitude improved goal-directed behavior in ACD would suggest the need to change strategies in abstinence therapies by weighting up reward rather than using other reinforcing contingencies (e.g., motivate drug users to attend treatment sessions regularly) [83,84,85,86]. In this regard, the fact that money availability can condition drug procurement [37,87] might mean that clinical interventions should focus on promoting the more immediate and reinforcing goal of social, family, and health rewards [17] because monetary reward cues could increase craving or drug urges, contributing to maintained drug use [22,88].

### Impact of monetary reward gradients on brain activity

Functional results confirm our second hypothesis because the activity of the prefrontal regions was negatively modulated by the increasing monetary reward magnitude in ACD compared to HC, but ACD increase the activation of other brain regions, such as the superior occipital cortex, which might counteract prefrontal differences with HC. The brain activation in the absence of performance differences between these same groups may be interpreted in several ways (see [41] for a discussion on similar effects), but the most frequent interpretation in drug addiction research would be to consider differences in patients’ brain activation as a vulnerability index for the cognitive dysfunction underlying the neuronal changes associated with lengthy drug use [89, 90,91,92]. The DLPFC plays an important role in integrating cognitive and motivational information processing [37,93,94], and previous studies using cognitive tasks have shown its involvement in reward modulation [95–99]. The interaction effect obtained in the DLPFC shows a difference between the groups’ BOLD signal in relation to their sensitivity to reward gradients. Thus, the reduced activation in the DLPFC during goal-directed behavior associated with achieving higher monetary rewards in ACD agrees with addiction features that compromise cognitive control and reduce reward sensitivity associated with drug-related changes and frontal lobe functioning and top-down processes [53,37,87]; although we observed increased activation in occipital regions in the ACD group. We suggest that reward may modulate different neural pathways from those shown to be modulated by monetary gradients in ACD compared to HC. We observed an interaction effect in the superior occipital cortex which could be interpreted as an attentional reactivity to reward that allowed functional compensation. In fact, previous studies evaluating the involvement of cocaine addiction in cognitive control functions such as attention [100] and working memory [31] found that, compared to HC, ACD showed hyperactivity in brain areas such as the parietal and occipital cortex. This result was interpreted as a greater recruitment of the visual attention network to support sustained visual processing, in order to compensate for the deficit in executive functions. In addition, previous studies using control attention tasks with monetary contingencies have related the enhancement of the bilateral occipital cortex to visual spatial attention [101,102], suggesting that the superior occipital cortex is an important area involved in attentional orientation, depending on the motivational meaning of the stimulus.

### Brain connectivity between left DLPFC and left putamen

We confirmed our third hypothesis. The DLPFC region that showed a reduced activation in response to the parametric increase in the monetary reward in ACD showed a greater connectivity with the left putamen in ACD than in HC. DLPFC-striatum connectivity has been described in classic accounts of motivation system anatomy as an associative network involved in goal-directed instrumental behavior sensitive to changes in the outcome of the action; that is, DLPFC motivational and cognitive information integration appears to rely on interactions with the striatum [103]. Although no previous studies have investigated the connectivity in the frontrostriatal network related to reward contingencies in cocaine addicts, Konova et al. [32] and Tau et al. [33] related the response to money in the striatum to impaired involvement of brain regions during task performance. Specifically, Konova et al. [32] associated putamen hyperactivity during a parametric increase in the monetary reward on a drug Stroop task with a general ventromedial prefrontal deactivation across reward conditions, which was interpreted as a compensatory mechanism necessary to maintain similar levels of performance. In our case, ACD showed an increased connectivity between the DLPFC and the putamen for higher reward gradients, which could represent the manifestation of the engagement of the frontostriatal circuits to support goal-directed behaviors in more effortful conditions, as previously suggested [33].

### Impact of craving and dependence severity on dorsal striatum modulation by reward

We partly confirmed our fourth hypothesis because regression results showed that high scores on self-reported craving were related to reduced activation of the left caudate during parametric increases in the monetary reward; however, high scores on self-reported dependence severity were related to enhanced activation of the right pallidum. Regarding the former, previous studies exploring cocaine craving observed that caudate activation increased during higher self-reported craving [22,88,104]. However, we should take into account that the methodology employed in these studies was quite different from ours because these paradigms induced craving by showing a cocaine video, and then they evaluated the relationship between self-reported craving at that moment and brain activity. By contrast, we used an interference Stroop condition in which participants were requested to involve cognitive-control processes to obtain money, whereas craving reported by the cocaine addicts refers to what they felt during the past two weeks. We suggest that left caudate hypoactivation could be related to poor cognitive control associated with craving according to previous studies [68]. Regarding addiction severity, taking into account the pallidum implication in the habitual behavior [105] and the drug-seeking habit [106–109], its involvement in increasing reward processing may be identifying individual differences in disease severity.

### Limitations

Our methodological approach has some limitations. We applied an fMRI block design, which entails problems such as habituation and anticipation and facilitates the application of strategic factors related to stimulus processing. To avoid these effects, we included no-conflict trials among the conflict ones (an approximate ratio of 1:4 for no-conflict: conflict trials). Although ROI is one of the most commonly used approaches because it is reliable and appropriate when studies have specific a priori hypotheses, it is limited by the constricted areas explored and the susceptibility of seed positioning variations. Our data cannot address the causal relationship between neural imaging measurements and behavioral improvement because we did not find a correlation between them. The present study does not include other psychometric psychological assessments, such as impulsivity or compulsivity, which are related to a neurobehavioral pattern of addiction. Finally, as in most studies with human clinical groups, it is not possible to address the etiology of these activity pattern differences; that is, we cannot say whether the differential brain recruitment patterns reported above pre-date and facilitate cocaine consumption, or whether they arose as a consequence of prolonged cocaine use.

## Supporting Information

S1 Fig Scatterplots displaying the partial correlation between the Self-reported interest for reward magnitudes (x axis) and RT and SDrt (y axis). The y axis values represent tenths of second; SDrt, reaction time Standard Deviation; RT, reaction time; HC, Healthy Control; ACD, Abstinent Cocaine Dependent; €, euro.
(DOC)

## References

[1] Volkow ND, Wang G-J, Fowler JS, Tomasi D. Addiction circuitry in the human brain. Annu Rev Pharmacol Toxicol [Internet]. 2012 1 [cited 2013 Oct 17];52(September):321–36. Available from: http://www.pubmedcentral.nih.gov/articlerender.fcgi?artid=3477468&tool=pmcentrez&rendertype=abstract 10.1146/annurev-pharmtox-010611-134625 21961707 PMC3477468

[2] Kalivas PW, Volkow ND. The neural basis of addiction: A pathology of motivation and choice. Am J Psychiatry. 2005;162(8):1403–13. 10.1176/appi.ajp.162.8.1403 16055761

[3] Bustamante J. C, Costumero V, Fuentes-Claramonte P, Rosell-Negre P, Ávila C. C OMPULSIVE D RUG S ELF -A DMINISTRATION: I NCENTIVE S ENSITIZATION AND L OSS OF T OP -D OWN C ONTROL IN C OCAINE A DDICTION. Cocaine Abus Pharmacol Treat Relapse Prev. 2012;In: Xi Chu(Substance Abuse Assessment, Interventions and Treatment; Public Health in the 21st Century):7x10 (NBC–R); ISBN: 978–1–61942–202–5.

[4] Robinson TE, Berridge KC. Review. The incentive sensitization theory of addiction: some current issues. Philos Trans R Soc Lond B Biol Sci [Internet]. 2008 10 12 [cited 2013 Oct 17];363(1507):3137–46. Available from: http://www.pubmedcentral.nih.gov/articlerender.fcgi?artid=2607325&tool=pmcentrez&rendertype=abstract 10.1098/rstb.2008.0093 18640920 PMC2607325

[5] Robinson TE, Berridge KC. Addiction. Annu Rev Psychol [Internet]. 2003 1 [cited 2014 Jul 10];54:25–53. Available from: http://www.ncbi.nlm.nih.gov/pubmed/12185211 10.1146/annurev.psych.54.101601.145237 12185211

[6] Aarts E, van Holstein M, Cools R. Striatal Dopamine and the Interface between Motivation and Cognition. Front Psychol [Internet]. 2011 1 [cited 2014 Sep 11];2(July):163 Available from: http://www.pubmedcentral.nih.gov/articlerender.fcgi?artid=3139101&tool=pmcentrez&rendertype=abstract 10.3389/fpsyg.2011.00163 21808629 PMC3139101

[7] Braver TS, Krug MK, Chiew KS, Kool W, Westbrook JA, Clement NJ, et al Mechanisms of motivation-cognition interaction: challenges and opportunities. Cogn Affect Behav Neurosci. 2014;14(2):443–72. 10.3758/s13415-014-0300-0 24920442 PMC4986920

[8] Jimura K, Locke HS, Braver TS. Prefrontal cortex mediation of cognitive enhancement in rewarding motivational contexts. Proc Natl Acad Sci U S A [Internet]. 2010 5 11 [cited 2014 Oct 5];107(19):8871–6. Available from: http://www.pubmedcentral.nih.gov/articlerender.fcgi?artid=2889311&tool=pmcentrez&rendertype=abstract 10.1073/pnas.1002007107 20421489 PMC2889311

[9] Arias-Carrión O, Stamelou M, Murillo-Rodríguez E, Menéndez-González M, Pöppel E. Dopaminergic reward system: a short integrative review. Int Arch Med. 2010;3:24 10.1186/1755-7682-3-24 20925949 PMC2958859

[10] Wise R a. Dopamine, learning and motivation. Nat Rev Neurosci. 2004;5(6):483–94. 10.1038/nrn1406 15152198

[11] Aarts E, Roelofs A, Franke B, Rijpkema M, Fernández G, Helmich RC, et al Striatal dopamine mediates the interface between motivational and cognitive control in humans: evidence from genetic imaging. Neuropsychopharmacology [Internet]. 2010 8 [cited 2014 Aug 16];35(9):1943–51. Available from: http://www.pubmedcentral.nih.gov/articlerender.fcgi?artid=3055632&tool=pmcentrez&rendertype=abstract 10.1038/npp.2010.68 20463658 PMC3055632

[12] Bustamante J-C, Barrós-Loscertales A, Costumero V, Fuentes-Claramonte P, Rosell-Negre P, Ventura-Campos N, et al Abstinence duration modulates striatal functioning during monetary reward processing in cocaine patients. Addict Biol [Internet]. 2013 9 [cited 2014 Sep 11];19(5):885–94. Available from: http://www.ncbi.nlm.nih.gov/pubmed/23445167 10.1111/adb.12041 23445167

[13] D.J., Nutt A., Lingford-Hughes D., Erritzoe PS. The dopamine theory of addiction: 40 years of highs and lows. Nat Rev Neurosci. 2015;16 (5):p305–12. 10.1038/nrn3939 25873042

[14] Everitt BJ, Robbins TW. From the ventral to the dorsal striatum: Devolving views of their roles in drug addiction. Neurosci Biobehav Rev. Elsevier Ltd; 2013;37(9):1946–54. 23438892 10.1016/j.neubiorev.2013.02.010

[15] Fowler JS, Volkow ND, Kassed C a, Chang L. Imaging the addicted human brain. Sci Pract Perspect [Internet]. 2007 4;3(2):4–16. Available from: http://www.pubmedcentral.nih.gov/articlerender.fcgi?artid=2851068&tool=pmcentrez&rendertype=abstract 17514067 10.1151/spp07324 PMC2851068

[16] Garavan H, Hester R. The role of cognitive control in cocaine dependence. Neuropsychol Rev [Internet]. 2007 9 [cited 2014 Sep 11];17(3):337–45. Available from: http://www.ncbi.nlm.nih.gov/pubmed/17680368 10.1007/s11065-007-9034-x 17680368

[17] Garavan H, Weierstall K. The neurobiology of reward and cognitive control systems and their role in incentivizing health behavior. Prev Med (Baltim). Elsevier Inc.; 2012;55(SUPPL.):S17–23. 10.1016/j.ypmed.2012.05.018 22683229

[18] Goldstein RZ, Craig a DB, Bechara A, Garavan H, Childress AR, Paulus MP, et al The neurocircuitry of impaired insight in drug addiction. Trends Cogn Sci [Internet]. 2009 9 [cited 2013 Oct 17];13(9):372–80. Available from: http://www.pubmedcentral.nih.gov/articlerender.fcgi?artid=2844118&tool=pmcentrez&rendertype=abstract 10.1016/j.tics.2009.06.004 19716751 PMC2844118

[19] Moeller SJ, Tomasi D, Honorio J, Volkow ND, Goldstein RZ. Dopaminergic involvement during mental fatigue in health and cocaine addiction. Transl Psychiatry [Internet]. Nature Publishing Group; 2012 1 [cited 2014 Sep 2];2(10):e176 Available from: http://www.pubmedcentral.nih.gov/articlerender.fcgi?artid=3565817&tool=pmcentrez&rendertype=abstract 23092980 10.1038/tp.2012.110 PMC3565817

[20] Yager LM, Garcia AF, Wunsch AM, Ferguson SM. The ins and outs of the striatum: Role in drug addiction. Neuroscience. IBRO; 2015;301:529–41. 10.1016/j.neuroscience.2015.06.033 26116518 PMC4523218

[21] Goldstein RZ, Volkow ND. Dysfunction of the prefrontal cortex in addiction: neuroimaging findings and clinical implications. Nat Rev Neurosci [Internet]. Nature Publishing Group; 2011 11 [cited 2013 Oct 17];12(11):652–69. Available from: http://www.pubmedcentral.nih.gov/articlerender.fcgi?artid=3462342&tool=pmcentrez&rendertype=abstract 10.1038/nrn3119 22011681 PMC3462342

[22] Garavan H, Pankiewicz J, Bloom a, Cho JK, Sperry L, Ross TJ, et al Cue-induced cocaine craving: neuroanatomical specificity for drug users and drug stimuli. Am J Psychiatry [Internet]. 2000 11;157(11):1789–98. Available from: http://www.ncbi.nlm.nih.gov/pubmed/11058476 10.1176/appi.ajp.157.11.1789 11058476

[23] Dunning JP, Parvaz M a, Hajcak G, Maloney T, Alia-Klein N, Woicik P a, et al Motivated attention to cocaine and emotional cues in abstinent and current cocaine users—an ERP study. Eur J Neurosci [Internet]. 2011 5 [cited 2014 Sep 4];33(9):1716–23. Available from: http://www.pubmedcentral.nih.gov/articlerender.fcgi?artid=3086977&tool=pmcentrez&rendertype=abstract 10.1111/j.1460-9568.2011.07663.x 21450043 PMC3086977

[24] Wilcox CE, Teshiba TM, Merideth F, Ling J, Mayer AR. Enhanced cue reactivity and fronto-striatal functional connectivity in cocaine use disorders. Drug Alcohol Depend. Elsevier Ireland Ltd; 2011;115(1–2):137–44. 10.1016/j.drugalcdep.2011.01.009 21466926 PMC3090708

[25] Jasinska AJ, Stein E a, Kaiser J, Naumer MJ, Yalachkov Y. Factors modulating neural reactivity to drug cues in addiction: a survey of human neuroimaging studies. Neurosci Biobehav Rev [Internet]. Elsevier Ltd; 2014 1 [cited 2014 Aug 12];38:1–16. Available from: http://www.ncbi.nlm.nih.gov/pubmed/24211373 10.1016/j.neubiorev.2013.10.013 24211373 PMC3913480

[26] Modesto-Lowe V, Burleson J a, Hersh D, Bauer LO, Kranzler HR. Effects of naltrexone on cue-elicited craving for alcohol and cocaine. Drug Alcohol Depend. 1997;49(1):9–16. 9476694 10.1016/s0376-8716(97)00134-8

[27] Colzato LS, van den Wildenberg WPM, Hommel B. Impaired inhibitory control in recreational cocaine users. PLoS One [Internet]. 2007 1 [cited 2014 Sep 11];2(11):e1143 Available from: http://www.pubmedcentral.nih.gov/articlerender.fcgi?artid=2065840&tool=pmcentrez&rendertype=abstract 10.1371/journal.pone.0001143 17989775 PMC2065840

[28] Fillmore MT, Rush CR. Impaired inhibitory control of behavior in chronic cocaine users. Drug Alcohol Depend. 2002;66(3):265–73. 12062461 10.1016/s0376-8716(01)00206-x

[29] Hester R, Garavan H. Executive dysfunction in cocaine addiction: evidence for discordant frontal, cingulate, and cerebellar activity. J Neurosci [Internet]. 2004 12 8 [cited 2013 Oct 17];24(49):11017–22. Available from: http://www.ncbi.nlm.nih.gov/pubmed/15590917 10.1523/JNEUROSCI.3321-04.2004 15590917 PMC6730277

[30] Ruiz MJ, Paolieri D, Colzato LS, Bajo MT. Chronic and recreational use of cocaine is associated with a vulnerability to semantic interference. Psychopharmacology (Berl). 2014;232(10):1717–26. 25413897 10.1007/s00213-014-3806-9

[31] Tomasi D, Goldstein RZ, Telang F, Maloney T, Alia-Klein N, Caparelli EC, et al Widespread disruption in brain activation patterns to a working memory task during cocaine abstinence. Brain Res [Internet]. 2007 9 26 [cited 2014 Sep 11];1171:83–92. Available from: http://www.pubmedcentral.nih.gov/articlerender.fcgi?artid=2048813&tool=pmcentrez&rendertype=abstract 10.1016/j.brainres.2007.06.102 17765877 PMC2048813

[32] Konova AB, Moeller SJ, Tomasi D, Parvaz M a, Alia-Klein N, Volkow ND, et al Structural and behavioral correlates of abnormal encoding of money value in the sensorimotor striatum in cocaine addiction. Eur J Neurosci [Internet]. 2012 10 [cited 2013 Oct 21];36(7):2979–88. Available from: http://www.pubmedcentral.nih.gov/articlerender.fcgi?artid=3463641&tool=pmcentrez&rendertype=abstract 10.1111/j.1460-9568.2012.08211.x 22775285 PMC3463641

[33] Tau GZ, Marsh R, Wang Z, Torres-Sanchez T, Graniello B, Hao X, et al Neural correlates of reward-based spatial learning in persons with cocaine dependence. Neuropsychopharmacology. Nature Publishing Group; 2014;39(3):545–55. 10.1038/npp.2013.189 23917430 PMC3895231

[34] Vadhan NP, Hart CL, Haney M, van Gorp WG, Foltin RW. Decision-making in long-term cocaine users: Effects of a cash monetary contingency on Gambling task performance. Drug Alcohol Depend. 2009;102(1–3):95–101. 10.1016/j.drugalcdep.2009.02.003 19346083 PMC2694492

[35] Engelmann JB, Damaraju E, Padmala S, Pessoa L. Combined effects of attention and motivation on visual task performance: transient and sustained motivational effects. Front Hum Neurosci [Internet]. 2009 1 [cited 2014 Sep 11];3(March):4 Available from: http://www.pubmedcentral.nih.gov/articlerender.fcgi?artid=2679199&tool=pmcentrez&rendertype=abstract 10.3389/neuro.09.004.2009 19434242 PMC2679199

[36] Guitart-Masip M, Huys QJM, Fuentemilla L, Dayan P, Duzel E, Dolan RJ. Go and no-go learning in reward and punishment: interactions between affect and effect. Neuroimage [Internet]. Elsevier Inc.; 2012 8 1 [cited 2014 Jul 9];62(1):154–66. Available from: http://www.pubmedcentral.nih.gov/articlerender.fcgi?artid=3387384&tool=pmcentrez&rendertype=abstract 10.1016/j.neuroimage.2012.04.024 22548809 PMC3387384

[37] Goldstein RZ, Alia-klein N, Tomasi D, Zhang L, Cottone L a, Maloney T, et al Is Decreased Prefrontal Cortical Sensitivity to Monetary and Self-Control in Cocaine Addiction? Psychiatry Interpers Biol Process. 2007;(January):43–51. 10.1176/appi.ajp.164.1.43 PMC2435056 17202543

[38] Kampman KM, Volpicelli JR, Mcginnis DE, Alterman AI, Weinrieb RM, D’Angelo L, et al Reliability and validity of the cocaine selective severity assessment. Addict Behav. 1998;23(4):449–61. 9698974 10.1016/s0306-4603(98)00011-2

[39] González-Sáiz F, Domingo-Salvany A, Barrio G, Sánchez-Niubó A, Brugal M T, de la Fuente L, Alonso J, Severity of Dependence Scale as a Diagnostic Tool for Heroin and Cocaine Dependence. Eur Addict Res 2009;15:87–93 10.1159/000189787 19142008

[40] Muñoz García M a., Martínez J a., Tejero A, Cepeda-Benito A. Development of the brief Spanish cocaine craving questionnaire-general. Psicothema. 2008;20(4):545–50. 18940049

[41] Barrós-Loscertales A, Bustamante JC, Ventura-Campos N, Llopis JJ, Parcet MA, Ávila C. Lower activation in the right frontoparietal network during a counting Stroop task in a cocaine-dependent group. Psychiatry Res—Neuroimaging. Elsevier Ireland Ltd; 2011;194(2):111–8. 10.1016/j.pscychresns.2011.05.001 21958514

[42] MacLeod CM. Half a century of research on the Stroop effect: an integrative review. Psychol Bull. 1991;109(2):163–203. 2034749 10.1037/0033-2909.109.2.163

[43] Adams ZW, Roberts WM, Milich R, Fillmore MT. Does response variability predict distractibility among adults with attention-deficit/hyperactivity disorder? Psychol Assess. 2011;23(2):427–36. 10.1037/a0022112 21443365 PMC3115498

[44] Carmona S, Hoekzema E, Ramos-Quiroga JA, Richarte V, Canals C, Bosch R, et al Response inhibition and reward anticipation in medication-na??ve adults with attention-deficit/hyperactivity disorder: A within-subject case-control neuroimaging study. Hum Brain Mapp. 2012;33(10):2350–61. 10.1002/hbm.21368 21826761 PMC6870239

[45] Logan GD, Zbrodoff NJ. When it helps to be misled: Facilitative effects of increasing the frequency of conflicting stimuli in a Stroop-like task. Mem Cognit. 1979;7(3):166–74.

[46] Logan GD, Cowan WB. On the ability to inhibit thought and action: A theory of an act of control. Psychol Rev. 1984;91(3):295–327. 10.1037/a0035230 24490789

[47] Panadero a., Castellanos MCC, Tudela P. Unconscious context-specific proportion congruency effect in a stroop-like task. Conscious Cogn. Elsevier Inc.; 2015;31(2015):35–45. 25460239 10.1016/j.concog.2014.09.016

[48] Friston KJ, Holmes a P, Poline JB, Grasby PJ, Williams SC, Frackowiak RS, et al Analysis of fMRI time-series revisited. NeuroImage. 1995 p. 45–53. 10.1006/nimg.1995.1007 9343589

[49] Weiland BJ, Welsh RC, Yau WYW, Zucker RA, Zubieta JK, Heitzeg MM. Accumbens functional connectivity during reward mediates sensation-seeking and alcohol use in high-risk youth. Drug Alcohol Depend [Internet]. Elsevier Ireland Ltd; 2013;128(1–2):130–9. 10.1016/j.drugalcdep.2012.08.019 22958950 PMC3546225

[50] Fleming SM, Huijgen J, Dolan RJ. Prefrontal contributions to metacognition in perceptual decision making. J Neurosci [Internet]. 2012;32(18):6117–25. Available from: http://www.jneurosci.org.gate1.inist.fr/content/32/18/6117.full 10.1523/JNEUROSCI.6489-11.2012 22553018 PMC3359781

[51] McLaren DG, Ries ML, Xu G, Johnson SC. A generalized form of context-dependent psychophysiological interactions (gPPI): A comparison to standard approaches. Neuroimage [Internet]. Elsevier B.V.; 2012;61(4):1277–86. 10.1016/j.neuroimage.2012.03.068 22484411 PMC3376181

[52] Gitelman DR, Penny WD, Ashburner J, Friston KJ. Modeling regional and psychophysiologic interactions in fMRI: The importance of hemodynamic deconvolution. Neuroimage. 2003;19(1):200–7. 12781739 10.1016/s1053-8119(03)00058-2

[53] Moeller SJ, Honorio J, Tomasi D, Parvaz M a., Woicik P a., Volkow ND, et al Methylphenidate enhances executive function and optimizes prefrontal function in both health and cocaine addiction. Cereb Cortex. 2014;24(3):643–53. 10.1093/cercor/bhs345 23162047 PMC3920764

[54] Leung HC, Skudlarski P, Gatenby JC, Peterson BS, Gore JC. An event-related functional MRI study of the stroop color word interference task. Cereb Cortex. 2000;10(6):552–60. 10859133 10.1093/cercor/10.6.552

[55] Brewer J a., Worhunsky PD, Carroll KM, Rounsaville BJ, Potenza MN. Pretreatment Brain Activation During Stroop Task Is Associated with Outcomes in Cocaine-Dependent Patients. Biol Psychiatry. Society of Biological Psychiatry; 2008;64(11):998–1004. 10.1016/j.biopsych.2008.05.024 18635157 PMC2601637

[56] Moeller SJ, Froböse MI, Konova AB, Misyrlis M, Parvaz M a., Goldstein RZ, et al Common and distinct neural correlates of inhibitory dysregulation: Stroop fMRI study of cocaine addiction and intermittent explosive disorder. J Psychiatr Res. 2014;58:55–62. 10.1016/j.jpsychires.2014.07.016 25106072 PMC4163519

[57] Worhunsky PD, Stevens MC, Carroll KM, Rounsaville BJ, Calhoun VD, Pearlson GD, et al Functional Brain Networks Associated With Cognitive Control, Cocaine Dependence, and Treatment Outcome. Psychol Addict Behav. 2013;27(2):477–88. 10.1037/a0029092 22775772 PMC3743442

[58] Mitchell Marci R., Balodis Iris M., DeVito Elise E., Lacadie Cheryl M. JY, Scheinost Dustin, Constable R. Todd, Carroll Kathleen M. and MNP. A preliminary investigation of Stroop-related intrinsic connectivity in cocaine dependence: Associations with treatment outcomes. 2013;39(6):1–22. 10.3109/00952990.2013.841711 PMC3827911 24200209

[59] Moeller SJ, Maloney T, Parvaz M a, Alia-Klein N, Woicik P a, Telang F, et al Impaired insight in cocaine addiction: laboratory evidence and effects on cocaine-seeking behaviour. Brain [Internet]. 2010 5 [cited 2014 Sep 11];133(Pt 5):1484–93. Available from: http://www.pubmedcentral.nih.gov/articlerender.fcgi?artid=2912695&tool=pmcentrez&rendertype=abstract 10.1093/brain/awq066 20395264 PMC2912695

[60] Bolla K., Ernst M., Kiehl K., Mouratidis M., Eldreth D., Contoreggi C., Matochik J., Kurian V., Cadet J., Kimes A., Funderburk F. & London E. Prefrontal Cortical Dysfunction in Abstinent Cocaine Abusers. J Neuropsychiatry Clin Neurosci. 2004;16(4):456–64. 10.1176/appi.neuropsych.16.4.456 15616172 PMC2771441

[61] Liu X, Banich MT, Jacobson BL, Tanabe JL. Common and distinct neural substrates of attentional control in an integrated Simon and spatial Stroop task as assessed by event-related fMRI. Neuroimage. 2004;22(3):1097–106. 10.1016/j.neuroimage.2004.02.033 15219581

[62] Noudoost B, Moore T. Control of visual cortical signals by prefrontal dopamine. Nature. Nature Publishing Group; 2011;474(7351):372–5. 10.1038/nature09995 21572439 PMC3117113

[63] Squire RF, Noudoost B, Schafer RJ, Moore T. Prefrontal contributions to visual selective attention. Annu Rev Neurosci. 2013;36:451–66. 10.1146/annurev-neuro-062111-150439 23841841

[64] Maldjian J a., Laurienti PJ, Kraft R a., Burdette JH. An automated method for neuroanatomic and cytoarchitectonic atlas-based interrogation of fMRI data sets. Neuroimage. 2003;19(3):1233–9. 12880848 10.1016/s1053-8119(03)00169-1

[65] Ikemoto S, Bonci A. Neurocircuitry of drug reward. Neuropharmacology. Elsevier; 2014;76(PART B):329–41. 23664810 10.1016/j.neuropharm.2013.04.031 PMC3772961

[66] Berridge Kent C. & Kringelbach Morten L.. Affective neuroscience of pleasure: reward in humans and animals. 2008;199(3):457–80. 10.1007/s00213-008-1099-6 18311558 PMC3004012

[67] Aron AR. From reactive to proactive and selective control: developing a richer model for stopping inappropriate responses. Biol Psychiatry [Internet]. Elsevier Inc.; 2011 6 15 [cited 2014 Jul 15];69(12):e55–68. Available from: http://www.pubmedcentral.nih.gov/articlerender.fcgi?artid=3039712&tool=pmcentrez&rendertype=abstract 10.1016/j.biopsych.2010.07.024 20932513 PMC3039712

[68] Grahn J a., Parkinson J a., Owen AM. The cognitive functions of the caudate nucleus. Prog Neurobiol. 2008;86(3):141–55. 10.1016/j.pneurobio.2008.09.004 18824075

[69] Balleine BW, Delgado MR, Hikosaka O. The role of the dorsal striatum in reward and decision-making. J Neurosci. 2007;27(31):8161–5. 10.1523/JNEUROSCI.1554-07.2007 17670959 PMC6673072

[70] Cools R. The cost of dopamine for dynamic cognitive control. Curr Opin Behav Sci. Elsevier Ltd; 2015;4:1–8.

[71] Volkow ND, Fowler JS, Wang G-J. The addicted human brain viewed in the light of imaging studies: brain circuits and treatment strategies. Neuropharmacology [Internet]. 2004 1 [cited 2014 Sep 9];47 Suppl 1:3–13. Available from: http://www.ncbi.nlm.nih.gov/pubmed/15464121 15464121 10.1016/j.neuropharm.2004.07.019

[72] Noël X, Brevers D, Bechara A. A neurocognitive approach to understanding the neurobiology of addiction. Curr Opin Neurobiol [Internet]. 2013 8 [cited 2013 Oct 17];23(4):632–8. Available from: http://www.ncbi.nlm.nih.gov/pubmed/23395462 10.1016/j.conb.2013.01.018 23395462 PMC3670974

[73] Crunelle CL, Veltman DJ, Booij J, Emmerik-van Oortmerssen K, van den Brink W. Substrates of neuropsychological functioning in stimulant dependence: a review of functional neuroimaging research. Brain Behav [Internet]. 2012 7 [cited 2014 Sep 11];2(4):499–523. Available from: http://www.pubmedcentral.nih.gov/articlerender.fcgi?artid=3432971&tool=pmcentrez&rendertype=abstract 10.1002/brb3.65 22950052 PMC3432971

[74] Poldrack R a. Region of interest analysis for fMRI. Soc Cogn Affect Neurosci [Internet]. 2007 3 [cited 2013 Oct 17];2(1):67–70. Available from: http://www.pubmedcentral.nih.gov/articlerender.fcgi?artid=2555436&tool=pmcentrez&rendertype=abstract 10.1093/scan/nsm006 18985121 PMC2555436

[75] Kriegeskorte N, Simmons WK, Bellgowan PSF, Baker CI. Circular analysis in systems neuroscience–the dangers of double dipping Supplementary Discussion A policy for noncircular analysis. 2009;1–30. 10.1038/nn.2303 PMC2841687 19396166

[76] Ashburner J, Chen C, Moran R, Henson R, Glauche V, Phillips C. SPM8 Manual The FIL Methods Group (and honorary members).

[77] Preacher, K. J. (2002, May). Calculation for the test of the difference between two independent correlation coefficients [Computer software]. Available from http://quantpsy.org.

[78] Goldstein Rita Z., Ph.D.*, a, Parvaz Muhammad A., M.S.a, b, Maloney Thomas, Ph.D.a Alia-Klein N, Ph.D.a, Woicik Patricia A., Ph.D.a, Telang Frank, M.D.a, Wang Gene-Jack MD., and Volkow Nora D. MD. Compromised sensitivity to monetary reward in current cocaine users: an ERP study. Changes. 2008;45(5):705–13. 10.1111/j.1469-8986.2008.00670.x PMC2574641 18513362

[79] Ahmed S.H., Kenny P.J., Koob G.F., Markou A., 2002 Neurobiological evidence for hedonic allostasis associated with escalating cocaine use. Nat.Neurosci. 5, 625–626. 10.1038/nn872 12055635

[80] Asensio S, Romero MJ, Palau C, Sanchez A, Senabre I, Morales JL, et al Altered neural response of the appetitive emotional system in cocaine addiction: an fMRI Study. Addict Biol [Internet]. 2010 10 [cited 2013 Oct 17];15(4):504–16. Available from: http://www.ncbi.nlm.nih.gov/pubmed/20579005 10.1111/j.1369-1600.2010.00230.x 20579005

[81] Hyatt CJ, Assaf M, Muska CE, Rosen RI, Thomas AD, Johnson MR, et al Reward-Related Dorsal Striatal Activity Differences between Former and Current Cocaine Dependent Individuals during an Interactive Competitive Game. 2012;7(5):1–15. 10.1371/journal.pone.0034917 PMC3351439 22606228

[82] Jia Z, Worhunsky PD, Carroll KM, Rounsaville BJ, Michael C, Pearlson GD, et al An initial study of neural responses to monetary. 2012;70(6):553–60. 10.1016/j.biopsych.2011.05.008 PMC3162064 21704307

[83] Stitzer M. L., Polk T., Bowles S., & Kosten T. (2010). Drug users’ adherence to a 6-month vaccination protocol: Effects of motivational incentives. Drug and Alcohol Dependence, 107(1), 76–79. 10.1016/j.drugalcdep.2009.09.006 19828264 PMC2815120

[84] Rogers RE, Higgins ST, Silverman K, Thomas CS, Badger J, Bigelow G, et al. drug Related Activities among Illicit Drug Abusers. 2010;22(4):544–50. 10.1037/0893-164X.22.4.544 PMC2825151 19071979

[85] Hester R, Bell RP, Foxe JJ, Garavan H. The influence of monetary punishment on cognitive control in abstinent cocaine-users. Drug Alcohol Depend [Internet]. Elsevier Ireland Ltd; 2013 11 1 [cited 2014 Sep 11];133(1):86–93. Available from: http://www.ncbi.nlm.nih.gov/pubmed/23791040 10.1016/j.drugalcdep.2013.05.027 23791040 PMC3786058

[86] Petry N.M., Martin B., 2002 Low-cost contingency management for treating cocaine- and opioid-abusing methadone patients. J. Consult. Clin. Psychol. 70, 398–405. 11952198 10.1037//0022-006x.70.2.398

[87] Goldstein RZ, Tomasi D, Alia-Klein N, Cottone L a, Zhang L, Telang F, et al Subjective sensitivity to monetary gradients is associated with frontolimbic activation to reward in cocaine abusers. Drug Alcohol Depend [Internet]. 2007 3 16 [cited 2013 Oct 17];87(2–3):233–40. Available from: http://www.pubmedcentral.nih.gov/articlerender.fcgi?artid=2435043&tool=pmcentrez&rendertype=abstract 10.1016/j.drugalcdep.2006.08.022 16997508 PMC2435043

[88] Volkow ND, Wang G, Telang F, Fowler JS, Logan J, Childress A, et al Cocaine Cues and Dopamine in Dorsal Striatum: Mechanism of Craving in Cocaine Addiction. 2006;26(24):6583–8. 10.1523/JNEUROSCI.1544-06.2006 16775146 PMC6674019

[89] Goldstein R.Z., Volkow N.D., Wang G.J., Fowler J.S., Rajaram S., 2001 Addiction changes orbitofrontal gyrus function: involvement in response inhibition. Neuroreport 12, 2595–2599. 11496155 10.1097/00001756-200108080-00060 PMC1201131

[90] Bolla K., Ernst M., Kiehl K., Mouratidis M., Eldreth D., Contoreggi C., Matochik J., Kurian V., Cadet J., Kimes A., Funderburk F., London E., 2004 Prefrontal cortical dysfunction in abstinent cocaine abusers. The Journal of Neuropsychiatry and Clinical Neurosciences 16, 456–464. 10.1176/appi.neuropsych.16.4.456 15616172 PMC2771441

[91] Li C.-S.R., Huang C., Yan P., Baghwagar Z., Milivojevic V., Sinha R., 2008 Neural correlates of impulse control during Stop signal inhibition in cocaine-dependent men. Neuropsychopharmacology 33, 1798–1806. 10.1038/sj.npp.1301568 17895916 PMC2731999

[92] Hester R., Nestor L., Garavan H., 2009 Impaired error awareness and anterior cingulate cortex hypoactivity in chronic cannabis users. Neuropsychopharmacology 34, 2450–2458. 10.1038/npp.2009.67 19553917 PMC2743772

[93] Ichihara-Takeda S, Funahashi S. Activity of primate orbitofrontal and dorsolateral prefrontal neurons: effect of reward schedule on task-related activity. J Cogn Neurosci [Internet]. 2008 4;20(4):563–79. Available from: http://www.ncbi.nlm.nih.gov/pubmed/18052781 10.1162/jocn.2008.20047 18052781

[94] Watanabe M, Sakagami M. Integration of cognitive and motivational context information in the primate prefrontal cortex. Cereb Cortex [Internet]. 2007 9 [cited 2014 Oct 22];17 Suppl 1:i101–9. Available from: http://www.ncbi.nlm.nih.gov/pubmed/17725993 17725993 10.1093/cercor/bhm067

[95] Krebs RM, Boehler CN, Egner T, Woldorff MG. The neural underpinnings of how reward associations can both guide and misguide attention. J Neurosci [Internet]. 2011 6 29 [cited 2014 Sep 11];31(26):9752–9. Available from: http://www.pubmedcentral.nih.gov/articlerender.fcgi?artid=3142621&tool=pmcentrez&rendertype=abstract 10.1523/JNEUROSCI.0732-11.2011 21715640 PMC3142621

[96] Gilbert AM, Fiez J a. Integrating rewards and cognition in the frontal cortex. Cogn Affect Behav Neurosci. 2004;4(4):540–52. 15849896 10.3758/cabn.4.4.540

[97] Pochon JB, Levy R, Fossati P, Lehericy S, Poline JB, Pillon B, et al The neural system that bridges reward and cognition in humans: an fMRI study. Proc Natl Acad Sci U S A. 2002;99(8):5669–74. 10.1073/pnas.082111099 11960021 PMC122829

[98] Staudinger MR, Erk S, Walter H. Dorsolateral prefrontal cortex modulates striatal reward encoding during reappraisal of reward anticipation. Cereb Cortex. 2011 11;21(11):2578–88. 10.1093/cercor/bhr041 21459835

[99] Stoppel CM, Boehler CN, Strumpf H, Heinze HJ, Hopf JM, Schoenfeld MA. Neural processing of reward magnitude under varying attentional demands. Brain Res. Elsevier B.V.; 2011;1383:218–29. 10.1016/j.brainres.2011.01.095 21295019

[100] Tomasi D, Goldstein RZ, Telang F, Maloney T, Alia-Klein N, Caparelli EC, et al Thalamo-cortical dysfunction in cocaine abusers: Implications in attention and perception. Psychiatry Res—Neuroimaging. 2007;155(3):189–201. 10.1016/j.pscychresns.2007.03.002 PMC2265105 17582746

[101] Small DM, Gitelman D, Simmons K, Bloise SM, Parrish T, Mesulam MM. Monetary incentives enhance processing in brain regions mediating top-down control of attention. Cereb Cortex. 2005;15(12):1855–65. 10.1093/cercor/bhi063 15746002

[102] Ivanov I, Liu X, Clerkin S, Schulz K, Friston K, Newcorn JH, et al Effects of motivation on reward and attentional networks: An fMRI study. Brain Behav. 2012;2(6):741–53. 10.1002/brb3.80 23170237 PMC3500461

[103] Cools R, Sheridan M, Jacobs E, D’Esposito M. Impulsive Personality Predicts Dopamine-Dependent Changes in Frontostriatal Activity during Component Processes of Working Memory. J Neurosci [Internet]. 2007;27(20):5506–14. 10.1523/JNEUROSCI.0601-07.2007 17507572 PMC6672352

[104] Sinha R, Lacadie C, Skudlarski P, Fulbright RK, Rounsaville BJ, Kosten TR, et al Neural activity associated with stress-induced cocaine craving: A functional magnetic resonance imaging study. Psychopharmacology (Berl). 2005;183(2):171–80. 16163517 10.1007/s00213-005-0147-8

[105] Tricomi E., Balleine B. W., & O’Doherty J. P. (2009). A specific role for posterior dorsolateral striatum in human habit learning. European Journal of Neuroscience, 29(11), 2225–2232. 10.1111/j.1460-9568.2009.06796.x 19490086 PMC2758609

[106] Stefanik MT, Kupchik YM, Brown RM, Kalivas PW. Optogenetic evidence that pallidal projections, not nigral projections, from the nucleus accumbens core are necessary for reinstating cocaine seeking. J Neurosci. 2013;33(34):13654–62. 10.1523/JNEUROSCI.1570-13.2013 23966687 PMC3755713

[107] Tang DW, Fellows LK, Small DM, Dagher a. Food and drug cues activate similar brain regions: a meta-analysis of functional MRI studies. Physiol Behav [Internet]. Elsevier Inc.; 2012 6 6 [cited 2014 Aug 26];106(3):317–24. Available from: http://www.ncbi.nlm.nih.gov/pubmed/22450260 10.1016/j.physbeh.2012.03.009 22450260

[108] Root DH, Fabbricatore AT, Ma S, Barker DJ, West MO. Rapid phasic activity of ventral pallidum neurons during cocaine self-adminstration. Synapse. 2011;64(9):704–13. 10.1002/syn.20792 PMC2904430 20340176

[109] McFarland K, Kalivas PW. The circuitry mediating cocaine-induced reinstatement of drug-seeking behavior. J Neurosci. 2001;21(21):8655–63. 11606653 10.1523/JNEUROSCI.21-21-08655.2001 PMC6762812

