# Supplementary material for: Reward Contingencies Improve Goal-Directed Behavior by Enhancing Posterior Brain Attentional Regions and Increasing Corticostriatal Connectivity in Cocaine Addicts
Source: PLoS One. 2016 Dec 1;11(12):e0167400. doi: 10.1371/journal.pone.0167400 (PMC5131954; doi:10.1371/journal.pone.0167400)
Supplement: S1 Fig — The y axis values represent tenths of second; SDrt, reaction time Standard Deviation; RT, reaction time; HC, Healthy Control; ACD, Abstinent Cocaine Dependent; €, euro. (DOC) [file pone.0167400.s001.doc]

**SUPPLEMENTAL INFORMATION**

**Reward contingencies improve goal-directed behavior by enhancing posterior brain attentional regions and increasing corticostriatal connectivity in cocaine addicts**

Patricia Rosell-Negre^a^, Juan-Carlos Bustamante^b^, Paola Fuentes-Claramonte^a,c^,

Víctor Costumero^a^, Juan-José Llopis-Llacer^d^, Alfonso Barrós-Loscertales^a^*

**ADDITIONAL FIGURE**

**Behavioral results**

**S1 Fig.** **Scatterplots displaying the partial correlation between the Self-reported interest for reward magnitudes (x axis) and RT and SDrt (y axis).**


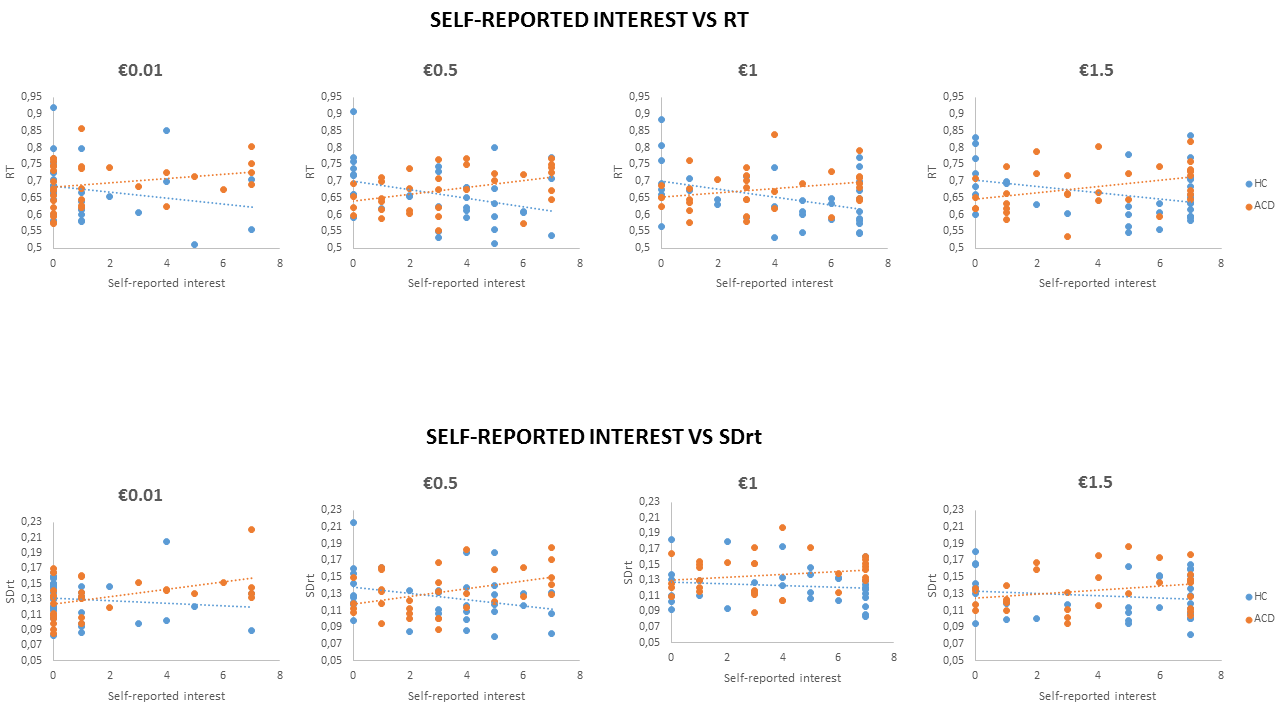


The y axis values represent tenths of second; SDrt, reaction time Standard Deviation; RT, reaction time; HC, Healthy Control; ACD, Abstinent Cocaine Dependent; €, euro.
